# Th17-related cytokines: new players in the control of chronic intestinal inflammation

**DOI:** 10.1186/1741-7015-9-122

**Published:** 2011-11-15

**Authors:** Ivan Monteleone, Francesco Pallone, Giovanni Monteleone

**Affiliations:** 1 Dipartimento di Medicina Interna, Università Tor Vergata, Via Montpellier, 1, 00133 Rome, Italy

## Abstract

Crohn's disease (CD) and ulcerative colitis (UC), the main forms of inflammatory bowel diseases (IBD) in man, are thought to be caused by an excessive and poorly controlled immune response that is directed against components of the normal microflora. The exact sequence of events by which this pathological process is triggered and maintained is not fully understood, but studies in experimental models of IBD and data emerging from recent clinical trials indicate that T cell-derived cytokines are crucial mediators of the tissue damage. Although CD and UC have been traditionally considered two typical examples of T helper (Th)1 or Th2-associated disease respectively, it is now known that CD- and UC-related inflammation is also marked by enhanced production of cytokines made by a distinct subset of Th cells, termed Th17 cells. Th17 cytokines can have both tissue-protective and inflammatory effects in the gut and there is evidence that Th17 cells can alter their cytokine program according to the stimuli received and convert into Th1-producing cells. These novel findings have contributed to advancing our understanding of mechanisms of gut tissue damage and open new avenues for development of therapeutic strategies in IBD.

## Background

Crohn's disease (CD) and ulcerative colitis (UC), as the main inflammatory bowel diseases (IBD) in man, are chronic and relapsing inflammatory disorders [1,2]. The etiology of IBD is still unknown, but it is believed that genetic and environmental factors interact to promote an excessive and poorly controlled mucosal inflammatory response directed against components of the luminal microflora [1,2]. Studies in experimental models also indicate that IBD-related tissue damage results from a dynamic interplay between immune and non-immune cells and that cytokines are crucial mediators of this cross-talk [1,2]. Analysis of the cytokine profile in IBD tissue has however shown that CD and UC are immunologically distinct. In CD there is predominance of T helper (Th)1-related cytokines, such as IL-12 and interferon IFN-γ, while in UC there is more IL-5 and IL-13, two Th2-associated cytokines [3-6]. Nonetheless, the demonstration that both anti-IL-12/p40 and anti-IFN-γ antibodies were only partially effective in patients with active CD [7-9] suggests that CD mucosal inflammation may be driven by additional mediators other than Th1 cytokines. Indeed, it is now well-known that CD and UC gut mucosa is heavily infiltrated with another subset of Th cells, termed Th17 cells, which produce a distinct array of cytokines [10-12]. In this article we review the available data supporting the role of Th17 cells in the modulation of gut inflammation and discuss whether and how Th17 cytokine-based therapy can enter into the therapeutic armamentarium of IBD.

### Th17-related cytokines are produced in excess in CD and UC tissue

**Figure 1:**
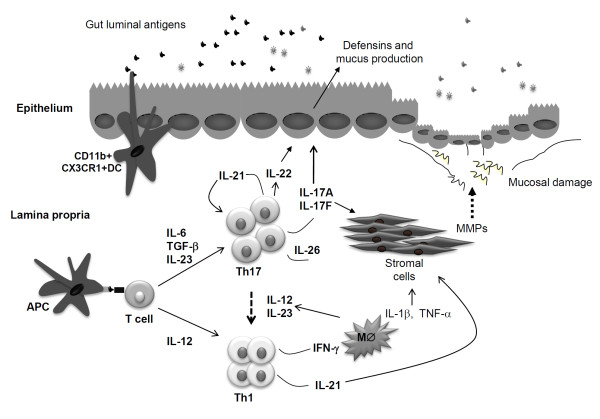
**The scheme illustrates some of the inflammatory and anti-inflammatory pathways activated by Th17 cytokines in the gut**. Antigen presenting cells (APC), such as dendritic cells and macrophages, respond to luminal antigens by producing cytokines, which promote the differentiation of naïve T helper (Th) cells in Th1 or Th17 cells. Th17 cells are, however, not stable and can convert to Th1 cells if they are stimulated by macrophage-derived (MØ) IL-12 and IL-23. In the gut of IBD patients, activated Th17 cells produce IL-17 (both A and F), IL-22, IL-26 and to a lesser extent IL-21, which is in contrast made preferentially by Th1 cells. IL-17 and IL-21 can induce stromal cells to secrete extracellular matrix-degrading proteases, such as matrix metalloproteinases (MMPs). IL-17A and IL-22 can exert anti-inflammatory effects due to their ability to stimulate anti-microbial peptides (that is defensins) and mucus secretion by epithelial cells. IFN-γ, a cytokine made by Th1 cells, can act on MØ and stimulate the production of other inflammatory cytokines, such as IL-1β and TNFα, which cooperate with Th17-type cytokines in promoting MMPs production by stromal cells.

Th17 cells produce IL-17, also termed IL-17A, IL-17F, IL-21, IL-22, and IL-26 [13,14]. Th17 cells can also make IL-9 [15,16]. However, analysis at the single cell-level has revealed that not all Th17 cells secrete all these cytokines, probably reflecting the heterogeneity of this cell subset. In mice, transforming growth factor β1 (TGF-β1) and IL-6 induce naïve T cells to express the transcription factors retinoic acid-related orphan receptor (ROR) γt and RORα and differentiate to Th17 cells [17,18] (Figure 1). An optimal expansion and maintenance of Th17 cell response requires activation of the transcription factor Stat3 and IL-23 [19]. By contrast, it is not yet fully understood how human Th17 cells differentiate *in vivo*, even though *in vitro *studies indicate that activation of CD4+ cells in the presence of IL-1β and IL-6 or IL-1β or IL-23 alone triggers IL-17A and IL-17F secretion [20,21]. IL-17A, IL-17F, IL-22 and IL-26 are all highly produced in the inflamed gut of patients with CD and patients with UC [10,12,22-24]. Moreover, IBD genome-wide association studies and candidate gene studies showed that polymorphisms of Th17-related genes, such as *Stat3 *or *IL-23R*, associate with IBD, thus supporting the involvement of the Th17 pathway into IBD pathogenesis [25-27]. Furthermore, elevated levels of CCL20, a chemoattractant for CCR6+ Th17 cells [28], have been documented in IBD mucosa, where CCL20 production seems to be positively regulated by IL-21, another Th17-related cytokine [29-31]. IL-21 is over-produced in the intestine of IBD patients, but the vast majority of IL-21-producing CD4+ T cells co-express IFN-γ and not IL-17A, thus suggesting that Th1 and not Th17 cells are major sources of IL-21 in the human gut [32,33] (Figure 1). This idea is supported by the fact that blockade of endogenous IL-12 in cultures of CD mucosal cells reduces IL-21 production while activation of normal intestinal CD4+ T lymphocytes in the presence of IL-12 increases the number of IL-21-secreting Th1 cells [32].

### The functional plasticity of Th17 cells

Flow-cytometry analysis of mononuclear cells isolated from the gut of CD patients has shown that some of the IL-17A-producing cells are not typical Th17 cells, because they co-express IFN-γ [34]. The majority of IL-17/IFN-γ-producing cells express CD161, a well known marker of NK T cells recently identified on IL-17-producing memory T cells [34]. These cells do not appear to be stable because *in vitro *stimulation with IL-12 enhances the expression of T-bet and IFN-γ, and down-regulates RORγt and IL-17A [12,35]. These findings support the notion that cells of the Th17 lineage are plastic and can be converted into Th1-type cells if they receive appropriate stimuli, such as IL-12 [12,35] (Figure 1). Although IL-23 was originally identified as a factor necessary for expanding/maintaining Th17 cell responses [19] and it is known that variants of the IL-23R gene influence IL-22 secretion [36], recent studies have shown that IL-23 can also facilitate the deviation from a Th17 to a Th1 phenotype [37,38] (Figure 1). This is in line with the demonstration that some of the pathogenic effects of IL-23 in the gut are linked to the ability of this cytokine to turn on IFN-γ [37]. Switching from IL-17A to IFN-γ production occurs if Th17 cells are activated in a microenvironment devoid of TGF-β1, because TGF-β1 is needed to maintain IL-17A production by *in vitro*-generated Th17 cells [21,39]. Since in the gut of IBD patients, high Smad7 abrogates TGF-β1 activity [40] and mucosal inflammation is marked by high IL-12 and IL-23 [3,41], it is conceivable that IFN-γ-producing cells seen in IBD tissue originate in part from pre-existing IL-17A-expressing T cells.

In addition to CD4+ T cells, many other cell types can produce Th17-related cytokines. For example, in IBD tissue, IL-17A is made by T cells and CD68+ macrophages [10]. NKT cells, NK cells, CD8+ T cells and γδ T cells can also secrete IL-17A and IL-22 [34,42-44]. IL-17A can also be produced by innate lymphoid cells (ILCs) [45,46]. IL-23-responsive ILCs, which are negative for CD3, CD127 and CD56, infiltrate the gut of patients with CD and the inflamed colons of Rag-deficient mice infected with *Helicobacter Hepaticus *[46,47]. These cells express high levels of IL-23R and RORγt and produce IFN-γ, IL-17A and IL-22 in response to IL-23 [47]. Depletion of these cell types attenuates colitis in mice [47], thus emphasizing their pathogenic role in the gut.

### Th17 cytokines have both anti-inflammatory and inflammatory effects in the gut

The discovery that Th17 cytokines are over-produced in IBD has boosted intensive research aimed at elucidating the contribution of each of these molecules in the control of gut inflammation. What has become evident is that some Th17 cytokines have both proinflammatory and tissue-protective properties, mostly depending on the model where they are studied. For example, there is evidence that IL-17A-knockout mice are more susceptible than control wild-type mice to developing colitis induced by oral administration of dextran sulfate sodium (DSS) [48]. DSS delivered with drinking water to mice causes disruption of the epithelial layer, which is followed by translocation of luminal bacteria to the mucosa and acute inflammatory response marked by a massive infiltration of the mucosa with neutrophils and macrophages [49]. Therefore, in this model, the tissue protective effect of IL-17A could rely on the ability of the cytokine to induce the expression of claudins in intestinal epithelial cells and to stimulate mucin production thereby enhancing the intestinal barrier [50] (Figure 1). An anti-inflammatory effect of IL-17A has also been seen in the T cell-transfer colitis model [51]. Indeed, it was shown that adoptive transfer of IL-17A-deficient naïve CD4+ T cells to recipient immunodeficient mice results in severe colitis [51]. Transfer of IL-17 receptor (IL-17R)-deficient T cells to recipient mice induces the same aggressive disease, indicating that IL-17 exerts its protective effects directly on T cells [51]. The greater severity of colitis induced by transfer of IL-17A-deficient T cells is not due to their enhanced migratory and infiltration capacity, but is instead related to enhanced Th1 cell effector function, raising the possibility that the anti-inflammatory effect of IL-17A in this model relies on the inhibition of Th1 cell responses.

Like IL-17A knockout mice, mice lacking IL-22 develop severe colitis following oral DSS administration as compared to wild-type mice. This observation fits well with the demonstration that IL-22 enhances intestinal barrier integrity, a phenomenon which seems to be dependent on the ability of this cytokine to activate Stat3 [52]. IL-22 stimulates epithelial cell growth, goblet cell restitution and mucus and antimicrobial production, thus restricting the passage of luminal commensal flora and food antigens to the lamina propria [23,52] (Figure 1). IL-22-mediated protective effects are also seen in the T cell transfer colitis model [53].

In contrast, mice deficient in IL-17RA are largely protected against experimental colitis induced by intrarectal administration of trinitrobenzenesulfonic acid (TNBS), and administration of IL-17RA IgG1 fusion protein attenuates TNBS-colitis in wild-type mice [54]. The fact that IL-17RA mediates the functional activities of both IL-17A and IL-17F [55] together with the above protective effects of IL-17A suggest that IL-17F, and not IL-17A, is pathogenic in the gut. This idea is supported by the demonstration that mice deficient in IL-17F are largely resistant against DSS-colitis [48]. The mechanism by which IL-17F promotes gut inflammation is not yet known, but there is evidence that this cytokine can induce the synthesis of various molecules (that is, TNF, IL-1, IL-6 and chemokines) that amplify pathogenic responses in the intestine [13,14].

Despite the above data suggesting that IL-17A has anti-inflammatory effects in the gut, we cannot exclude the possibility that, under specific circumstances, this cytokine can cooperate with IL-17F and other Th17 or Th1 cytokines to expand the IBD-associated inflammatory response. This hypothesis is supported by studies performed by Leppkes and colleagues [56] showing that transfer of IL-17A-, IL-17F-, or IL-22-deficient T lymphocytes into RAG1-null mice induces severe colitis that is indistinguishable from that caused by wild-type cells. In contrast, transfer of RORγ-null T cells, which associates with no induction of IL-17 cytokines in the intestine, does not induce colitis [56]. Moreover, treatment of RAG1 mice that received IL-17F-null T cells with a neutralizing anti-IL-17A antibody suppresses disease [56].

Transfer of naïve CD8+ T cells into syngeneic RAG-deficient mice results in severe colitis, similar to that seen after transfer of naïve CD4+ T cells [57]. Analysis of CD8+ T cells in the mesenteric lymph nodes of such mice show the existence of IL-17A and IFN-γ- double-positive cells. Notably, transfer of naïve CD8+ T cells derived from either IL-17- or IFN-γ-knockout mice is associated with less severe colitis. Like naïve CD4+ cells lacking RORγt, CD4+ T cells deficient in Th1-related transcription factors (that is, Stat4 and T-bet) are unable to induce colitis when transferred to recipient mice [58,59]. Altogether these observations suggest that a mixture of both Th1 and Th17 cytokines are needed to promote pathology in the gut and that compounds interfering with both Th1 and Th17 cell activity could be useful to facilitate the resolution of the ongoing mucosal inflammation in IBD. In this context, a promising target could be IL-21, whose activity seems to be necessary for expanding both Th1 and Th17 cell responses in the gut [32,60]. Like human IBD, mice with acute DSS-colitis and TNBS-relapsing colitis produce elevated levels of IL-21, and administration of a neutralizing IL-21R fusion protein to DSS-treated mice attenuates the ongoing colitis and reduces the production of Th17-related cytokines [60]. IL-21-deficient mice are largely protected against DSS- and TNBS-colitis, and this protection is associated with a marked decrease in IL-17A and IL-17F, thus confirming the key role of IL-21 in sustaining Th17 immunity [60]. IL-21 exerts further biological functions that could contribute to its pro-inflammatory effect in the gut. For example, IL-21 inhibits the peripheral differentiation of regulatory T cells (Tregs) and makes CD4+ T cells resistant to Tregs-mediated immune suppression [61]. Like IL-17A, IL-21 stimulates stromal cells to produce tissue-degrading proteases [62] (Figure 1). Human intestinal epithelial cells express IL-21R and respond to IL-21 by up-regulating the secretion of the T cell chemoattractant macrophage inflammatory protein-3α [31]. It remains unknown, however, if this occurs also in mice, because no study has yet demonstrated that murine colonic epithelial cells express IL-21R. Finally, there is evidence that IL-21 enhances the expression of Th1-related transcription factors and IFN-γ in T and NK cells [63,64].

### Aryl hydrocarbon receptor signalling promotes IL-22 synthesis and attenuates gut inflammation

Th17 cells that have lost the ability to secrete IL-17A and turned on IFN-γ express high levels of aryl hydrocarbon receptor (AhR) [65,66], raising the possibility that AhR can control the activity of these cells. AhR is a transcription factor ubiquitously expressed in vertebrate cells and able to mediate a range of cellular events in response to halogenated aromatic hydrocarbons, non-halogenated polycyclic aromatic hydrocarbons, small synthetic compounds and natural chemicals, including derivatives of tryptophan, such as 6-formylindolo (3, 2-b) carbazole (Ficz) [67]. Activation of AhR results in enhanced production of Th17 cytokines, particularly IL-22, and reduction of Th1 and Th2 cytokine [65,68-72]. There is however evidence that AhR-deficient CD4+ T cells can be induced to differentiate along the Th17 pathway, thus suggesting that AhR is probably involved in the expansion, rather than induction, of Th17 cells [73]. In line with the above findings, we have recently shown that administration of Ficz to mice ameliorated TNBS-, relapsing DSS- and T cell-transfer colitis [74]. These data were paralleled by inhibition of Th1 cytokines and up-regulation of IL-22. Moreover, treatment of mice with an AhR antagonist reduced IL-22 production and enhanced the severity of TNBS-colitis [74]. Blockade of IL-22 with a neutralizing antibody reversed the therapeutic effect of Ficz in mice with TNBS-colitis, thus indicating that induction of IL-22 is one of the major mechanisms by which AhR signals control pathogenic responses in the gut [74].

Analysis of AhR expression in human IBD demonstrated a marked down-regulation in the inflamed tissue of CD patients as compared to uninvolved areas of the same patients, inflamed areas of UC patients and normal controls. Importantly, AhR content did not significantly differ between UC and normal control samples thus suggesting that the diminished expression of AhR in CD is not an epiphenomenon of the ongoing inflammation [74]. As in mice, treatment of human IBD mucosal cells with Ficz resulted in decreased IFN-γ expression and up-regulation of IL-22. Time-course studies showed, however, that suppression of Th1 cell response preceded IL-22 induction thus supporting the ability of AhR to control distinct pathways of mucosal damage and healing [74].

### Summary and future directions

The findings described in this article together with the beneficial effects of Th17-cytokine blockers in immune-mediated pathologies, such as psoriasis [75], suggest that targeting Th17 cytokines could be a rationale approach to dampen the detrimental inflammatory response in IBD. However, results of clinical trials of two different antibodies neutralizing the IL-23/p40 subunit were quite disappointing in CD [7,76] and a recent study has shown that blockade of IL-17A is not effective in CD [77]. These negative results are not, however, entirely surprising because, as pointed out in this article, the IBD-associated tissue-damaging immune response is driven by additional cytokines other than Th17-related molecules. If so, we can speculate that simultaneous neutralization of two or more of these molecules (for eample IFN-γ and IL-17A) could help manage the active phases of IBD patients.

## Conclusions

In recent years, progress in basic and translational research has led to a better understanding of the role of Th17 cytokines in the control of gut inflammation. What has become evident is that these molecules can have both inflammatory and anti-inflammatory effects in the gut and that some cytokines can cooperate with molecules produced by other cell subsets in amplifying inflammatory processes. The available data seem to suggest that compounds inhibiting some Th17-related cytokines (that is IL-17A, IL-22) could not be effective in IBD or could worsen the IBD course. By contrast, it is conceivable that blockers of IL-21 or IL-17F are more advantageous because these two cytokines sustain multiple inflammatory signals in the gut. However, given the clinical and biological heterogeneity of IBD, further experimentation will be necessary to establish whether IL-21 and IL-17F are produced at a high level during the various phases of the disease.

## Abbreviations

AhR: aryl hydrocarbon receptor; CD: Crohn's disease; DSS: dextran sulfate sodium; Ficz: 6-formylindolo (3: 2-b) carbazole; IBD: inflammatory bowel disease; IFN: interferon; IL: interleukin; ILC: innate lymphoid cells; IL-17R: interleukin-17 receptor; ROR: retinoic acid related orphan receptor; TGF: transforming growth factor; TNBS: trinitrobenzenesulfonic acid; Tregs: regulatory T cells; UC: ulcerative colitis.

## Competing interests

G.M. has filed a patent entitled 'Interleukin-21 (IL-21) binding proteins and methods of making and using same' (European Patent Application No.08425294.9).

## Authors' contributions

IM, FP and GM have contributed equally to drafting the manuscript and making the figure. All authors read and approved the final manuscript.

## Pre-publication history

The pre-publication history for this paper can be accessed here:

http://www.biomedcentral.com/1741-7015/9/122/prepub
